# Polycystic Ovary Syndrome Triggers Atrial Conduction Disorders: A Systematic Review and Meta-Analysis

**DOI:** 10.3390/ejihpe12070059

**Published:** 2022-07-13

**Authors:** Dimitrios V. Moysidis, Andreas S. Papazoglou, Christos Tsagkaris, Vasileios Oikonomou, Anna Loudovikou, Anastasios Kartas, Nikolaos Stalikas, Efstratios Karagiannidis, Mihnea-Alexandru Găman, Marios Papadakis, Chrysi Christodoulaki, Periklis Panagopoulos

**Affiliations:** 1 Faculty of Medicine, Aristotle University of Thessaloniki, 54124 Thessaloniki, Greece; aspapazo@auth.gr (A.S.P.); vasoiknik@auth.gr (V.O.); tkartas@gmail.com (A.K.); nstalik@gmail.com (N.S.); stratoskarag@gmail.com (E.K.); 2 Public Health and Policy Working Group, Stg European Student Think Tank, Postjeskade 29, 1058 DE Amsterdam, The Netherlands; chriss20x@gmail.com (C.T.); loudovikou.anna@gmail.com (A.L.); 3 Faculty of Medicine, “Carol Davila” University of Medicine and Pharmacy, 050474 Bucharest, Romania; 4 Center of Hematology and Bone Marrow Transplantation, Department of Hematology, Fundeni Clinical Institute, 022328 Bucharest, Romania; 5 Department of Surgery II, University Hospital Witten-Herdecke, Heusnerstrasse 40, University of Witten-Herdecke, 42283 Wuppertal, Germany; marios_papadakis@yahoo.gr; 6 Family Planning Unit, Third Department of Obstetrics and Gynaecology, Attiko Hospital, National and Kapodistrian University of Athens, 15772 Athens, Greece; christodoulakichr@hotmail.com (C.C.); paninosrafaela@yahoo.gr (P.P.)

**Keywords:** polycystic ovaries, atrial fibrillation, atrial conduction disorders, P-wave, atrial electromechanical delay, atrial arrhythmias, insulin resistance, systematic inflammation, ovulatory dysfunction

## Abstract

*Background:* Polycystic ovary syndrome (PCOS) is closely related to various adverse cardiovascular manifestations and increased cardiovascular risk. However, atrial fibrillation (AF) development and atrial conduction abnormalities have not been thoroughly studied in patients with PCOS. *Methods:* This meta-analysis (CRD42021261375) was conducted in accordance with the PRISMA guidelines. Our aim was to investigate associations between PCOS and disorders in atrial conduction parameters linked with an increased risk for AF occurrence. *Results:* Five cohort studies with aggregate data on 406 adult women (229 with PCOS and 177 age-matched without PCOS) were included in this analysis. Our results showed a significantly increased mean difference in P-wave maximum duration (+7.63 ± 7.07 msec; *p* < 0.01) and P-wave dispersion (+11.42 ± 5.22 msec; *p* = 0.03) of patients with PCOS compared to healthy women. The mean difference in P-wave minimum duration (−2.22 ± 2.68 msec; *p* = 0.11) did not reach the statistical threshold between the compared groups. Echocardiographic measurements of atrial electromechanical delay (AED) also indicated a statistically significant mean difference in favour of the PCOS group in all assessed parameters, except for atrial electromechanical coupling (PA) in the tricuspid annulus. Particularly, PCOS was associated with increased lateral PA, septal PA, inter- and intra-AED durations (mean difference: +17.31 ± 9.02 msec; *p* < 0.01, +11.63 ± 7.42 msec; *p* < 0.01, +15.31 ± 9.18 msec; *p* < 0.01, +9.31 ± 6.85 msec; *p* < 0.01, respectively). *Conclusions:* PCOS is strongly associated with alterations in several electrocardiographic and echocardiographic parameters indicating abnormal atrial conduction. Therefore, PCOS could be considered as a causal or triggering factor of AF. Larger studies are needed to confirm these results and investigate direct associations between PCOS and AF.

## 1. Introduction

Polycystic ovary syndrome (PCOS) is a common complex endocrine-metabolic disease affecting women of reproductive age, and is clinically characterized by ovulatory dysfunction, hyperandrogenism, infertility and increased cardiovascular risk [1,2,3]. PCOS is complicated by obesity, insulin resistance (IR), diabetes mellitus, dyslipidemia, and metabolic syndrome, caused by a genetic predisposition to abnormal-excessive steroidogenesis, irregular peripheral insulin sensitivity and lipid metabolism alterations [4,5,6,7,8,9]. Additionally, IR and the compensatory hyperinsulinemia have been blamed for the increased rates of hypertension in patients with PCOS due to hypertrophy of vascular smooth muscle cells and activation of the renin-angiotensin-aldosterone system [10]. These factors along with the endothelial dysfunction induced by elevated androgen levels and IR are responsible for the association of PCOS with the development of comorbid cardiovascular diseases [11,12,13].

Atrial fibrillation (AF) constitutes the most prevalent sustained arrhythmia in clinical practice, which consists of electrophysiological and electromechanical abnormalities which are caused by intra-atrial and inter-atrial conduction disorders [14,15]. Prolonged maximum P-wave duration (Pmax), increased P-wave dispersion (Pdis) and echocardiographic measurements of atrial electromechanical delay (AED) are all well-known electrophysiological markers indicating that atria prone to fibrillate [16]. According to recent studies, these parameters have been shown to independently predict the development of AF [17,18].

Despite being known for its adverse cardiovascular implications, PCOS has not been thoroughly investigated as a predictor of atrial conduction disorders and possibly AF occurrence. The aim of this study was, therefore, to review and meta-analyze up-to-date scientific data from patients with PCOS and healthy controls, in order to assess if PCOS could be considered as a direct causal factor of abnormal atrial conduction, thereby increasing the risk of AF triggering.

## 2. Materials and Methods

The present systematic review and meta-analysis was performed according to the Preferred Reporting Items for Systematic reviews and Meta-Analyses (PRISMA) guidelines [19]. Its predetermined research protocol has been registered a priori in the PROSPERO database (CRD42021261375).

### 2.1. Literature Search

A literature search was performed independently by two main reviewers (ASP and DVM) in PubMed (MEDLINE), Scopus, Web of Science, and Cochrane Library (CENTRAL) databases. The last search was performed on 7 June 2022. Basic keywords used in search strings were [“polycystic ovary syndrome” or PCOS] and [“atrial fibrillation” or AF or “atrial conduction disorders” or “P-wave” or “atrial arrhythmias”] in both free text and in Medical Subject Headings (MeSH) format. Additionally, the reference lists of the eligible studies were hand-searched to identify further potentially eligible papers not previously detected (snowball strategy).

### 2.2. Eligibility Criteria

Studies were included in this analysis if they were observational (prospective or retrospective), cohort or case-control studies reporting the difference in atrial conduction and electromechanical times among adult women (>18 years old) with or without PCOS. Eligible study populations should therefore consist of both: (1) an exposed group of women with PCOS, as defined according to the Rotterdam (histological or clinical) diagnostic criteria of PCOS [2], and (2) a non-exposed group of women without PCOS.

Studies were excluded if: (1) they were conference abstracts or letters; (2) they were published in a language other than English and German; (3) they were conducted in pediatric populations; (4) they did not report any effect measures [mean values (±standard deviations, SD)] for the outcomes of interest or if the study findings were unavailable; and (5) they a control group of women participants without PCOS, despite evaluating atrial conduction and electromechanical times in patients with PCOS.

### 2.3. Outcomes of Interest and Definitions

The selected studies should report evidence about the primary and/or the secondary outcomes of interest. The primary outcome of this analysis was the difference in electrocardiographic data concerning the P-wave maximum and minimum duration (P_max_ and P_min_, respectively), as well as the difference between the P_max_ and the P_min_, defined as P-wave dispersion (P_dis_) [20], among age-matched women with and without PCOS. To that end, a 12-lead ECG was obtained from each study participant of the selected studies with a standardized paper speed of 50 mm/s and signal size of 10 mm/mV, and all the aforementioned markers of atrial conduction have been recorded as mean ± SD milliseconds (msec), if possible.

The identification of between-group differences in atrial electromechanical delay (AED) echocardiographic measurements was the secondary outcome of this analysis. In particular, the following indicators of atrial conduction heterogeneity were obtained by tissue Doppler imaging and were documented as mean ± SD msec, where available:

1. the average time interval from onset of P-wave on surface ECG to the beginning of the late diastolic wave, (atrial electromechanical coupling, PA), which was obtained from:
  - the lateral mitral valve annulus (PA lateral),
  - the septal mitral valve annulus (PA septal),
  - and the right ventricular tricuspid annulus (PA tricuspid),
2. the difference between PA lateral and PA tricuspid (PA lateral—PA tricuspid), defined as inter-AED, and the difference between PA septum and PA tricuspid (PA septum—PA tricuspid), defined as intra-AED [16].

### 2.4. Data Extraction and Quality Assessment

Two main reviewers (DVM and ASP) independently undertook and completed both study selection and data extraction. After deduplication, the two independent reviewers screened all articles at the title and abstract level. Potentially eligible studies were further reviewed based on the full text. Discrepancies were resolved either by consensus or in consultation with a third reviewer (CT). The eligible studies were reviewed comprehensively to extract the predetermined data of interest. An electronic data extraction form (Excel) was used to record data on study design, sample size, population characteristics, utilized PCOS diagnostic criteria, endocrinological variables, and measured outcomes of interest, where available. The corresponding study authors were also contacted via email to obtain any significant missing data. The methodological quality of the included studies was independently assessed using the Newcastle-Ottawa scale [21], while the possibility of publication bias was visually assessed via the funnel plot method described by Egger and colleagues [22].

### 2.5. Data Synthesis and Analysis

The aforementioned endocrinological, electrocardiographic and echocardiographic data have been recorded for both women with and without PCOS. To allow for expected effect size dispersion between studies, random effects models and the inverse variance method were used to perform our meta-analysis according to the expected heterogeneity. In particular, random-effects models were utilized for the analyses, due to the interpreter-dependency which is usually the case in the interpretation of echocardiographic and electrocardiographic findings. Continuous variables were summarized as mean (±standard deviation; SD) and were analyzed by weighted mean differences, where applicable. Heterogeneity was tested with the Cochrane χ^2^ test and quantified by the I^2^ statistics and its 95% confidence intervals, with an I^2^ = 30–60% being considered as moderate. Revman 5.4. software was utilized for the statistical analysis.

## 3. Results

**Figure 1:**
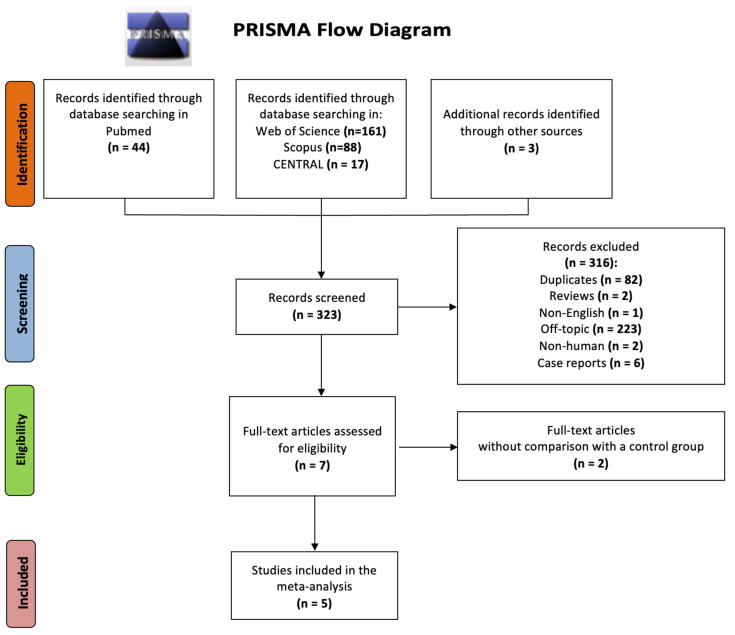
PRISMA flow diagram for study selection.

After an extensive literature search and study selection as presented in the PRISMA Flowchart (Figure 1), five cohort studies, investigating the predictive role of PCOS in terms of atrial conduction disorders, were included in our analysis. In total, 406 adult women were studied: 229 with PCOS and 177 age-matched without PCOS. The characteristics (study design, country, study sample size, PCOS diagnostic criteria, assessed outcomes, and score of Newcastle-Ottawa Scale) of the eligible studies are summarized in Table 1. In accordance with the quality evaluation criteria of the Newcastle-Ottawa scale, all included studies were characterized as “good”, fulfilling the predetermined Selection, Comparability, and Outcome/Exposure criteria: three or four stars in the Selection (S) domain AND 1 or 2 stars in Comparability (C) domain AND two or three stars in Outcome/Exposure (O-E) domain.

**Figure 2:**
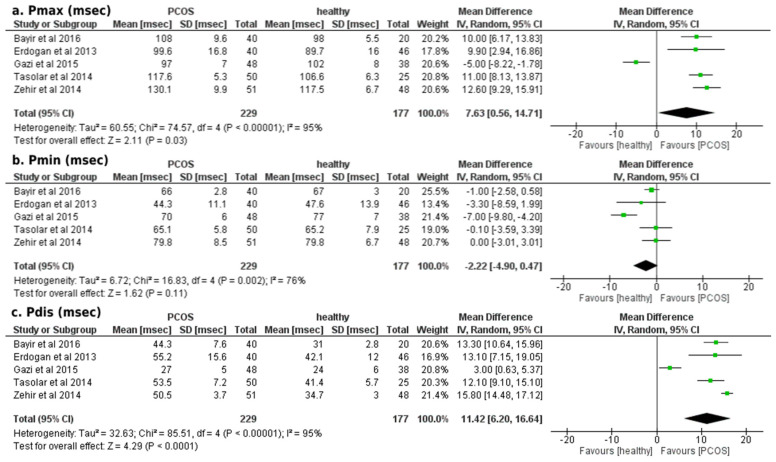
Forest plots of comparison for the primary outcome of the study (atrial conduction times, electrocardiographic findings): (**a**) Differences in P_max_ duration (msec) among women with and without PCOS, (**b**) Differences in P_min_ duration (msec) among women with and without PCOS and (**c**) Differences in P_dis_ duration (msec) among women with and without PCOS [23,24,25,26,27].

**Figure 3:**
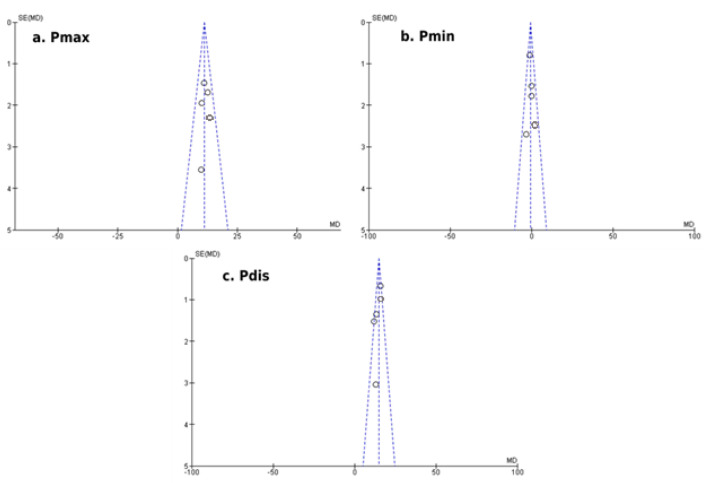
Egger’s funnel plots of comparison for the primary outcome of the study (atrial conduction times, electrocardiographic findings): (**a**) Differences in P_max_ duration (msec) among women with and without PCOS, (**b**) Differences in P_min_ duration (msec) among women with and without PCOS and (**c**) Differences in P_dis_ duration (msec) among women with and without PCOS.

Our results showed a significantly increased mean difference in P_max_ duration (+7.63 ± 7.07 msec; *p* < 0.01) and P_dis_ (+11.42 ± 5.22 msec; *p* = 0.03) of patients with PCOS compared to healthy participants, as illustrated in Figure 1. The mean difference in P_min_ duration (−2.22 ± 2.68 msec; *p* = 0.11) did not reach the statistical threshold between the two groups (Figure 2). Egger’s funnel plots for the primary outcome of our meta-analysis are depicted in Figure 3, demonstrating the potential risk of bias within the included studies.

**Figure 4:**
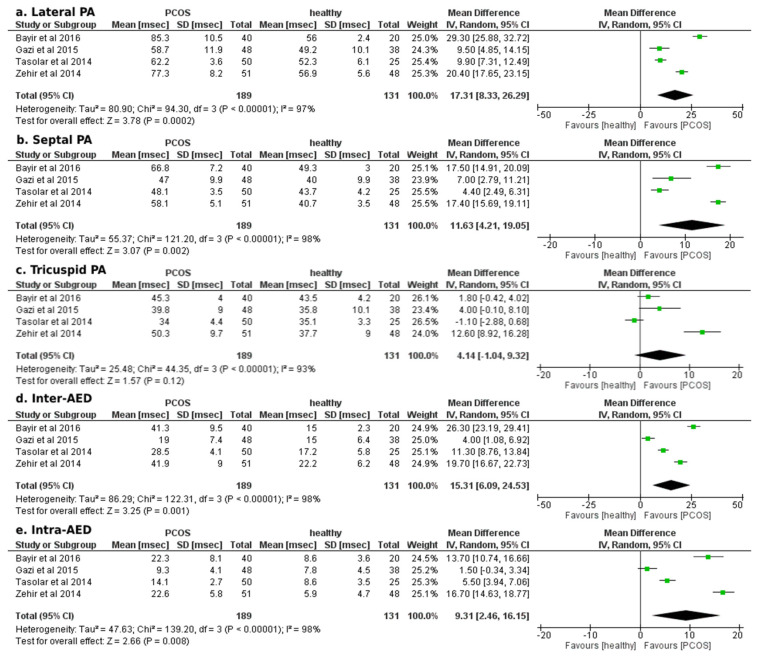
Forest plots of comparison for the secondary outcome of the study (atrial electromechanical delay, Doppler tissue echocardiographic findings): (**a**) Differences in lateral PA duration (msec) among women with and without PCOS, (**b**) Differences in septal PA duration (msec) among women with and without PCOS, (**c**) Differences in tricuspid PA duration (msec) among women with and without PCOS, (**d**) Differences in inter-atrial electromechanical delay (msec) among women with and without PCOS, and (**e**) Differences in intra-atrial electromechanical delay (msec) among women with and without PCOS [23,24,25,26,27].

Concerning the secondary outcome of echocardiographic measurements of AED, our analysis indicated a statistically significant mean difference in favour of the PCOS group in all assessed parameters of AED, except for the tricuspid PA (Figure 4). In particular, PCOS was associated with increased lateral PA, septal PA, inter- and intra-AED durations (mean difference: +17.31 ± 9.02 msec; *p* < 0.01, +11.63 ± 7.42 msec; *p* < 0.01, +15.31 ± 9.18 msec; *p* < 0.01, +9.31 ± 6.85 msec; *p* < 0.01, respectively).

When extracting the endocrinological variables evaluated in each study (Table 2), it was (qualitatively) shown that the homeostasis model assessment of IR was significantly higher among women with PCOS compared to controls in three out of four studies in which it was assessed, and was positively linked with prolonged inter-AED in two of them. A quantitative analysis of the studied endocrinological variables could not be performed due to the inconsistent presentation of the relevant variables across the eligible studies.

## 4. Discussion

Our meta-analysis of five cohorts with adult women with and without PCOS demonstrated that atrial conduction is influenced in PCOS, while the prevalence of atrial conduction and AED disorders is significantly higher in women with PCOS compared to healthy ones. PCOS seems to be a predictor of abnormal atrial conduction and, hence, could be considered as a pre-disposer for AF occurrence. Our findings yielded a strong, direct association between PCOS and increases in P_max_, P_dis_ and echocardiographic measurements of AED (lateral PA, septal PA, inter- and intra-AED durations). To our knowledge, our study is the first meta-analysis aiming to clarify these aspects, and therefore contributes not only to the identification of new possible causing and triggering factors of atrial conduction abnormalities and arrhythmias, but also to new interdisciplinary approaches to common gynecological diseases, such as PCOS.

The pathophysiology behind the effects of PCOS on electrocardiograms and the development of heterogenic atrial activity has not been sufficiently elucidated. PCOS is related to low-grade systematic inflammation and a higher risk of adverse cardiovascular events due to metabolic syndrome, hypertension, IR, obesity and dyslipidemia [11]. From an endocrinological point of view, insulin seems to be the main driver of cardiovascular disease risk in patients with PCOS. Of note, IR was assessed by a homeostasis model assessment of IR in four of the included studies, and was reported to be significantly higher in women with PCOS in three of them. IR and the compensatory hyperinsulinemia are considered per se as a potential mediator of excess ovarian androgen production, which modifies gonadal steroidogenesis process [28]. This contributes to the development of visceral adiposity, which further exacerbates obesity—a major predictor of AF occurrence and recurrence, maintaining this vicious cycle [29,30].

Metabolic syndrome, as expressed by obesity, dyslipidemia, hypertension and, primarily, IR, creates functional and structural vascular dysfunction, and causes inter- and intra-atrial conduction delays by stimulating interstitial fibrosis, worsening diastolic function and increasing the atrial size [31,32,33,34,35]. Apart from the well-known risk factors triggered by IR, hyperhomocysteinemia is also present in PCOS due to the dysregulation of enzymes participating in homocysteine metabolism [i.e., Methyltetrahydrofolate Reductase (MTHFR) and hepatic Cystathione β-Synthase (CBS)] [36]. Hyperhomocysteinemia induces increased inflammatory cytokine expression/activity, the injury of endothelial cells and proliferation of muscle cells, and has also been linked with increased risk for AF development and major adverse cardiovascular events [37]. Furthermore, a reasonable question arises: whether these comorbidities—known for their arrhythmogenic effects [38]—trigger AF, and PCOS is just a confounder and not the cause of the arrhythmia. Nonetheless, in four out of five included studies in our analysis, the two compared groups were, in general, well matched not only in terms of baseline characteristics but also in terms of other cardiovascular risk factors.

Moreover, two other mechanisms suggested as possible explanations for the increased rate of atrial conduction disorders in PCOS are systematic inflammation and autonomic dysfunction [39]. Multiple studies on inflammatory diseases have indicated structural and electrophysiological changes as a result of the systematic inflammation in the atrial myocardium [40,41,42]. Increased plasma volume, enhanced neurohormonal activation and ventricular diastolic dysfunction which accompany systematic inflammation might contribute to the left atrium enlargement and electrical instability. Despite all of this, the exact pathophysiologic mechanism behind the association of PCOS with abnormal atrial conduction remains unknown.

Furthermore, a direct cause-effect link between PCOS and atrial arrhythmias has not been yet established. Nevertheless, this can be hypothesized given the fact that atrial conduction disorders provide a suitable substrate for re-entry and thus constitute a precursor for AF development. A variety of studies showed that alterations in P-wave electrocardiographic parameters, mainly the increase in P_max_ and P_dis_, are simple predictive markers for the development and recurrence of idiopathic AF [17,43,44]. AED has been also linked to a higher risk of AF, as it has been proven to be longer in patients with AF than in controls [45]. On the other hand, these correlations between atrial conduction times and atrial arrhythmia occurrence have not been evaluated and validated in patients with PCOS to date. However, a recent large cohort study deriving results from a national registry robustly showed that women with PCOS were at a 2-fold greater risk than controls [46]. To the best of the authors’ knowledge, this was the first study to investigate and evince that PCOS appears to be an independent risk factor for AF.

This analysis should be interpreted in the context of some limitations. First and foremost, the five included studies are observational non-randomized-controlled trials, and are thus susceptible to inherent biases. Hence, the observational nature of the eligible studies, and the fact that they are single-centered with a relatively small number of patients, decreases the external validity of our study’s message. Additionally, the included studies are all restricted to a specific geographic area, which might reduce the generalizability of our analysis to other populations. Moreover, although our study highlighted a strong association between PCOS and atrial conduction abnormalities, it is not meant to establish a direct causal relationship between PCOS and AF. This is because the analyzed studies were not able to assess the development and long-term incidence of AF in PCOS patients and controls. Finally, emerging data suggest that PCOS is complicated with obesity, IR, low-grade systematic inflammation and the fibrosis process and, consequently, it could be considered as a confounding factor and not a direct predictor of atrial conduction disorders leading to AF occurrence.

Future studies need to address the aforementioned limited factors by means of randomized design and geographic diversity. It is also important to delve into the pathophysiology of this comorbidity and validate whether PCOS have a causal or cofounding association with atrial conduction disorders. This could lead to novel interdisciplinary approaches between cardiovascular and gynecological endocrinology research, enhance the collaboration between researchers of both fields, and ultimately raise awareness among concerned clinicians and patients.

## 5. Conclusions

PCOS is strongly associated with increases in P_max_, P_dis_, AED and electromechanical coupling parameters, indicating abnormal atrial conduction. The alterations in these electrocardiographic and echocardiographic parameters imply that PCOS could be a causing or triggering factor of AF. Larger and more representative studies are needed to confirm our results and to investigate direct associations between these two clinical entities.

## Figures and Tables

**Table 1:** Design and characteristics of the included studies.

First Author, Year	Study Type	Country	Sample Size, (N)	PCOS Diagnosis	Outcomes Assessed	Quality Score in NOS (S/C/O-E)
Bayir et al., 2016 [23]	prospective cohort	Turkey	N = 60: 40 adult women with PCOS and 20 age-matched without PCOS	Rotterdam 2003 criteria	ECG: P_max_, P_min_, and P_dis_ Doppler tissue echocardiography: Lateral PA, Septal PA, Tricuspid PA, Inter-AED, Intra-AED.	3/1/2
Gazi et al., 2015 [24]	prospective cohort	Turkey	N = 86: 48 adult women with PCOS and 38 age-matched without PCOS	Rotterdam 2003 criteria	ECG: P_max_, P_min_, and P_dis_ Doppler tissue echocardiography: Lateral PA, Septal PA, Tricuspid PA, Inter-AED, Intra-AED.	3/1/3
Tasolar et al., 2014 [25]	observational cohort	Turkey	N = 75: 50 women with PCOS (18–40 years of age) and 25 age-matched without PCOS	Rotterdam 2003 criteria	ECG: P_max_, P_min_, and P_dis_ Doppler tissue echocardiography: Lateral PA, Septal PA, Tricuspid PA, Inter-AED, Intra-AED.	3/1/3
Zehir et al., 2014 [26]	observational cohort	Turkey	N = 99: 51 adult women with PCOS and 48 age-matched without PCOS	Rotterdam 2003 criteria	ECG: P_max_, P_min_, and P_dis_ Doppler tissue echocardiography: Lateral PA, Septal PA, Tricuspid PA, Inter-AED, Intra-AED.	4/1/3
Erdogan et al., 2013 [27]	cross-sectional study	Turkey	N = 86: 40 women with PCOS (18–40 years of age) and 46 age-matched without PCOS	Rotterdam 2003 criteria	ECG: P_max_, P_min_, and P_dis_ Other echocardiographic measurements	3/1/3

Legend: PCOS, polycystic ovary syndrome; ECG, electrocardiogram; Pmax, P-wave maximum; Pmin, P-wave minimum; Pdis, P-wave dispersion; PA, atrial electromechanical coupling; AED, atrial electromechanical delay; NOS, Newcastle-Ottawa-Scale; S/C/O-E, Selection, Comparability, and Outcome/Exposure.

**Table 2:** Endocrinological variables assessed in each eligible study among women with and without PCOS.

Study	Endocrinological Variable Assessed	Women with PCOS	Women without PCOS	*p*-Value	Significant Association with Inter-AED
Bayir et al., 2016 [23]	Fasting glucose (mg/dL)	78.9 ± 5.8	79.0 ± 5.6	0.936	Ν/A
Gazi et al., 2015 [24]	FSH (mIU/mL) LH (mIU/mL) Estradiol (pg/mL) Testosterone (ng/dL) Fasting glucose (mg/dL) Fasting insulin (μIU/mL) Homeostasis model assessment of insulin resistance (IR)	5.07 (2.92–10.1) 6.62 (2.35–39.25) 43.2 ± 17.8 75.5 (14.7–314) 86 ± 12 15.28 ± 23.45 1.40 (0.37–36.15)	7.68 (2.02–19.10) 6.74 (2.03–19.47) 28.8 ± 11.3 17.2 (2.5–44) 87 ± 8 12.74 ± 17.57 1.44 (0.38–18.99)	0.001 0.442 0.001 0.001 0.945 0.627 0.659	n.s. n.s. n.s. *p* = 0.052, β-0.242 (univariate) n.s. n.s. n.s.
Tasolar et al., 2014 [25]	Estradiol (pg/mL) Testosterone (nmol/L) Fasting glucose (mmol/L) Fasting insulin (mU/L) Homeostasis model assessment of IR	67.1 ± 10.1 2.25 ± 0.48 4.2 ± 0.35 10 ± 0.7 1.90 ± 0.42	109.7 ± 8.3 1.17 ± 0.13 3.9 ± 0.2 5.6 ± 0.6 0.95 ± 0.12	<0.001 <0.001 n.s. <0.001 <0.001	r = −0.572, *p* < 0.001 n.s. r = −0.550, *p* < 0.001 r = 0.939, *p* < 0.001 r = 0.940, *p* < 0.001 β = 0.603, *p* < 0.001 (multivariate)
Zehir et al., 2014 [26]	FSH (mIU/mL) LH (mIU/mL) Estradiol (pg/mL) Testosterone (ng/dL) Fasting glucose (mg/dL) Prolactin(ng/mL) DHEA-S (mg/dL) Homeostasis model assessment of IR	5.4 ± 1.1 6.2 ± 2.0 60.8 ± 5.07 8.1 ± 7.4 82.3 ± 4.8 17.2 ± 1.4 293.2 ± 62.3 3.1 ± 0.7	5.5 ± 1.1 6.1 ± 1.0 58.9 ± 4.8 52.9 ± 6.1 86.3 ± 5.8 16.7 ± 1.2 245.0 ± 29.2 1.6 ± 0.4	n.s. n.s. n.s. <0.001 n.s. n.s. <0.001 <0.001	N/A N/A N/A n.s. N/A N/A n.s. r = 0.680, *p* < 0.001
Erdogan et al., 2013 [27]	Fasting glucose (mg/dL) Fasting insulin (mU/L) Homeostasis model assessment of IR	87 ± 6 19.8 ± 20.3 4.24 ± 4.17	88 ± 4 9.6 ± 3.1 2.07 ± 0.72	n.s. 0.014 0.013	N/A N/A N/A

## Data Availability

Not applicable.
